# Epigenomic reprogramming of therapy-resistant circulating tumor cells in colon cancer

**DOI:** 10.3389/fcell.2023.1291179

**Published:** 2023-12-21

**Authors:** Aida Bao-Caamano, Nicolás Costa-Fraga, Laure Cayrefourcq, Aitor Rodriguez-Casanova, Laura Muinelo-Romay, Rafael López-López, Catherine Alix-Panabières, Angel Díaz-Lagares

**Affiliations:** ^1^ Epigenomics Unit, Cancer Epigenomics, Translational Medical Oncology Group (ONCOMET), Health Research Institute of Santiago de Compostela (IDIS), University Clinical Hospital of Santiago (CHUS/SERGAS), Santiago de Compostela, Spain; ^2^ Universidade de Santiago de Compostela (USC), Santiago de Compostela, Spain; ^3^ Galician Precision Oncology Research Group (ONCOGAL), Medicine and Dentistry School, Universidade de Santiago de Compostela (USC), Santiago de Compostela, Spain; ^4^ Laboratory of Rare Human Circulating Cells–The Liquid Biopsy Lab, University Medical Center of Montpellier, Montpellier, France; ^5^ Centre for Ecological and Evolutionary Cancer Research, Maladies infectieuses et vecteurs: génétique, èvolution et contrôle, University of Montpellier, CNRS, IRD, Montpellier, France; ^6^ Roche-Chus Joint Unit, Translational Medical Oncology Group (ONCOMET), Health Research Institute of Santiago (IDIS), Santiago de Compostela, Spain; ^7^ Liquid Biopsy Analysis Unit, Translational Medical Oncology Group (ONCOMET), Health Research Institute of Santiago de Compostela (IDIS), Santiago de Compostela, Spain; ^8^ Centro de Investigación Biomédica en Red Cáncer (CIBERONC), ISCIII, Madrid, Spain; ^9^ Translational Medical Oncology Group (ONCOMET), Health Research Institute of Santiago de Compostela (IDIS), University Clinical Hospital of Santiago (CHUS/SERGAS), Santiago de Compostela, Spain; ^10^ European Liquid Biopsy Society (ELBS), Hamburg, Germany; ^11^ Department of Clinical Analysis, University Hospital Complex of Santiago de Compostela (CHUS), Santiago de Compostela, Spain

**Keywords:** Epigenomics, DNA methylation, metastasis-competent CTCs, colorectal cancer, biomarkers, therapeutic targets, therapy resistance

## Abstract

Therapy resistance is a major challenge in colorectal cancer management. Epigenetic changes, such as DNA methylation, in tumor cells are involved in the development of acquired resistance during treatment. Here, we characterized the DNA methylation landscape of colon circulating tumor cells (CTCs) during cancer progression and therapy resistance development. To this aim, we used nine permanent CTC lines that were derived from peripheral blood samples of a patient with metastatic colon cancer collected before treatment initiation (CTC-MCC-41) and during treatment and cancer progression (CTC-MCC-41.4 and CTC-MCC-41.5 [A-G]). We analyzed the DNA methylome of these nine CTC lines using EPIC arrays and also assessed the association between DNA methylation and gene expression profiles. We confirmed DNA methylation and gene expression results by pyrosequencing and RT-qPCR, respectively. The global DNA methylation profiles were different in the pre-treatment CTC line and in CTC lines derived during therapy resistance development. These resistant CTC lines were characterized by a more hypomethylated profile compared with the pre-treatment CTC line. Most of the observed DNA methylation differences were localized at CpG-poor regions and some in CpG islands, shore regions and promoters. We identified a distinctive DNA methylation signature that clearly differentiated the pre-treatment CTC line from the others. Of note, the genes involved in this signature were associated with cancer-relevant pathways, including PI3K/AKT, MAPK, Wnt signaling and metabolism. We identified several epigenetically deregulated genes associated with therapy resistance in CTCs, such as *AP2M1*. Our results bring new knowledge on the epigenomic landscape of therapy-resistant CTCs, providing novel mechanisms of resistance as well as potential biomarkers and therapeutic targets for advanced CRC management.

## 1 Introduction

Colorectal cancer (CRC) is the third most frequent cancer and the second leading cause of cancer-related mortality worldwide (Sung et al., 2021). Up to 50% of patients with CRC present metastases at diagnosis or develop metastases during the disease course (Biller and Schrag, 2021). Despite recent advances in treatment regimens, fluoropyrimidine-based chemotherapy continues to be frequently used for metastatic CRC (mCRC). However, tumors quickly fail to respond due to the development of resistance (Narayan et al., 2022).

Analysis of liquid biopsy analytes, such as circulating tumor cells (CTCs), has demonstrated clinical utility for the non-invasive evaluation of the therapy response. Indeed, CTC enumeration can provide information about CRC progression and therapy response (Aranda et al., 2020). Moreover, nine CTC lines generated from blood samples of a patient with mCRC in Alix-Panabières’ laboratory allow studying therapy-driven tumor changes (Soler et al., 2018). This series of nine colon CTC lines were established from peripheral blood samples collected from the same patient at different time-points during the disease course: i) before the first treatment (CTC-MCC-41 line) (Cayrefourcq et al., 2015) and ii) after treatment: CTC-MCC-41.4 line (after first-line FOLFIRI/bevacizumab and second-line FOLFOX/bevacizumab), and 7 cell lines (CTC-MCC-41.5 [A-G]) established from blood collected 1 week before the patient´s death. The cell lines CTC-MCC-41.5 [A-G] were originated from the same blood sample after proliferating *in vitro* independently at different time points and exhibiting different morphological features (Soler et al., 2018). Of note, the transcriptomic analysis of all these CTC lines revealed that treatment pressure can induce changes in their gene expression profiles associated with therapy resistance, indicating that the molecular profiling of these CTC lines is a relevant approach to study disease progression and discover new biomarkers of therapy resistance (Cayrefourcq et al., 2021).

Some studies demonstrated that CTCs are characterized by DNA methylation profile alterations that promote metastasis formation (Chimonidou et al., 2011; Gkountela et al., 2019). DNA methylation is based on the incorporation of a methyl group into the cytosine of cytosine-phosphate-guanine (CpG) dinucleotides. This epigenetic modification usually occurs in the context of high density CpG dinucleotides, called CpG islands (CpGIs). However, DNA methylation can also occur in regions with lower CpG density that flank CpGIs (CpG shores) (Rodriguez-Casanova et al., 2021). In cancer, aberrant DNA methylation in CpGIs and shores of promoters is usually associated with changes in gene expression, with a negative correlation between DNA methylation and gene expression (Diaz-Lagares et al., 2016; Tan et al., 2019). Of note, DNA methylation alterations in CRC are closely related to cancer progression and chemotherapy resistance (Moutinho et al., 2014; Diaz-Lagares et al., 2016). However, no study investigated whether DNA methylation changes in colorectal CTCs are associated with cancer progression and therapy resistance.

Therefore, in this work, we used a genome-wide DNA methylation analysis to compare the DNA methylation landscape of the previously described nine colon CTC lines with the aim of obtaining new biological information and identifying biomarkers and therapeutic targets linked to CRC progression and therapy resistance.

## 2 Materials and methods

### 2.1 Cancer cell lines

The nine colon CTC lines used in this work (CTC-MCC-41, CTC-MCC-41.4, CTC-MCC-41.5A, CTC-MCC-41.5B, CTC-MCC-41.5C, CTC-MCC-41.5D, CTC-MCC-41.5E, CTC-MCC-41.5F and CTC-MCC-41.5G) have been previously characterized (Cayrefourcq et al., 2015; Soler et al., 2018). All CTC lines were cultured at 37°C and 5% CO_2_ in ultra-low attachment flasks (Corning) with RPMI-1640 medium (Merck) supplemented with 10% fetal bovine serum (Merck), 1% penicillin/streptomycin solution (Gibco), 1% L-glutamine (Merck), 1% of Insulin-Transferrin-Selenium (ITS-G) (Gibco), basic human fibroblast growth factor (Miltenyi Biotec) at a final concentration of 10 ng/mL and human epidermal growth factor (Miltenyi Biotec) at a final concentration of 20 ng/mL.

### 2.2 DNA and RNA isolation from the nine CTC lines

Total genomic DNA was isolated from the nine CTC lines using a standard high salt method based on SDS/proteinase K. The isolated DNA was treated with RNase A (Qiagen) following the manufacturer’s recommendations and stored at −80°C until analysis. All DNA samples were quantified with the Qubit 4 fluorometric method (Invitrogen) using the Qubit dsDNA BR (Broad Range) Assay Kit (Invitrogen). Total RNA was isolated from the CTC lines using TRIzol (Invitrogen) according to the manufacturer’s protocol, quantified using a NanoDrop One spectrophotometer (Thermo Scientific), and stored at −80°C until analysis.

### 2.3 Genome-wide DNA methylation analysis by EPIC arrays

Total genomic DNA (500 ng) from three passages of the nine CTC lines was treated with sodium bisulfite using the EZ DNA Methylation kit (Zymo Research). Following the manufacturer’s protocol, the bisulfite-converted DNA was hybridized using the Infinium MethylationEPIC (EPIC) array that targets >850,000 CpG sites in the human genome (Moran et al., 2016). After methylation values were obtained in a HiScan System (Illumina), data were processed using RnBeads 2.0 (Muller et al., 2019). Raw intensity data (IDAT) files were imported into RnBeads 2.0 for quality control and preprocessing. First, the greedycut algorithm was used to filter out low-quality probes. Probes overlapping with single nucleotide polymorphisms and probes with sequences that mapped to multiple genomic locations (cross-reactive) were removed. Raw IDAT files were normalized using the beta-mixture quantile (BMIQ) method. DNA methylation was represented as the average β-value that was calculated as the ratio of the fluorescence signal intensity of the methylated probe to the signals of all (methylated and unmethylated) probes. The average β-values were used to calculate the mean DNA methylation difference between groups. Hierarchical linear models performed with RnBeads 2.0 were used to determine the DNA methylation differences between groups. *p*-values were corrected for multiple testing (FDR) using the Benjamini–Hochberg method and a *p*-value <0.05 was selected for significance. Heatmaps of β-values were generated using unsupervised hierarchical clustering and the Complex Heatmap package. GeneCodis was used for Gene Ontology (GO) enrichment and Panther pathway analyses (Tabas-Madrid et al., 2012).

### 2.4 Locus-specific DNA methylation analysis by pyrosequencing

Total genomic DNA (500 ng) from three passages of the nine CTC cell lines was treated with sodium bisulfite with the EZ DNA Methylation kit (Zymo Research) following the manufacturer’s recommendations. For pyrosequencing, primer sequences (Supplementary Table S1) were designed with PyroMark Assay Design 2.0 (Qiagen). Standard PCRs were carried out with bisulfite-converted genomic DNA. PCR products were analyzed on 2% agarose gels before pyrosequencing. A PyroMark Q24 Vacuum Workstation was used for the immobilization and preparation of PCR products. Pyrosequencing reactions were performed using a PyroMark Gold Q24 Reagent Kit (Qiagen) following the manufacturer’s instructions. DNA methylation values were obtained using the PyroMark Q24 software 2.0 (Qiagen). Human methylated and non-methylated DNA samples (Zymo Research) were used as positive and negative control, respectively. Water was used as no-template control.

### 2.5 Gene expression analysis

Microarray expression data (Affymetrix HG-U133P) of the nine CTC cell lines were from a previous study by Cayrefourcq et al. (Cayrefourcq et al., 2021). For reverse transcription-quantitative PCR (RT-qPCR), the isolated RNA was first treated with DNase I using the Turbo DNA-free Kit (Invitrogen) according to the manufacturer’s recommendations. Next, 1–2 μg of RNA was retrotranscribed using the SuperScript First-Strand Synthesis System for RT‒PCR (Invitrogen). Reactions were performed in triplicate on a StepOne Plus system (Applied Biosystems) using 25–200 ng cDNA, 10 μL Power SYBR Green PCR Master Mix (Applied Biosystems) and 0.3 μL of the 10 μM specific primers in a final volume of 20 μL. Results were normalized to the expression level of β2-microglobulin (endogenous control) in each sample. The primers used are listed in Supplementary Table S2.

### 2.6 Statistical analysis

Hierarchical linear models performed with RnBeads 2.0 were used to determine the DNA methylation differences between groups. *p*-values were corrected for multiple testing (FDR) using the Benjamini–Hochberg method and a *p*-value <0.05 was selected for significance. To compare DNA methylation data and gene expression values obtained by pyrosequencing and RT-qPCR, the data distribution normality was tested using the Kolmogorov-Smirnov test followed by the Mann‒Whitney *U* test or the Student’s t-test. The GraphPad Prism 8.0 software and the R statistical environment (version 4.2.0) were used for statistical analyses. All *p*-values were calculated with two-tailed tests and were considered significant when the *p*-value was <0.05.

## 3 Results

### 3.1 DNA methylation differences between the pre- and post-treatment CTC lines

**FIGURE 1:**
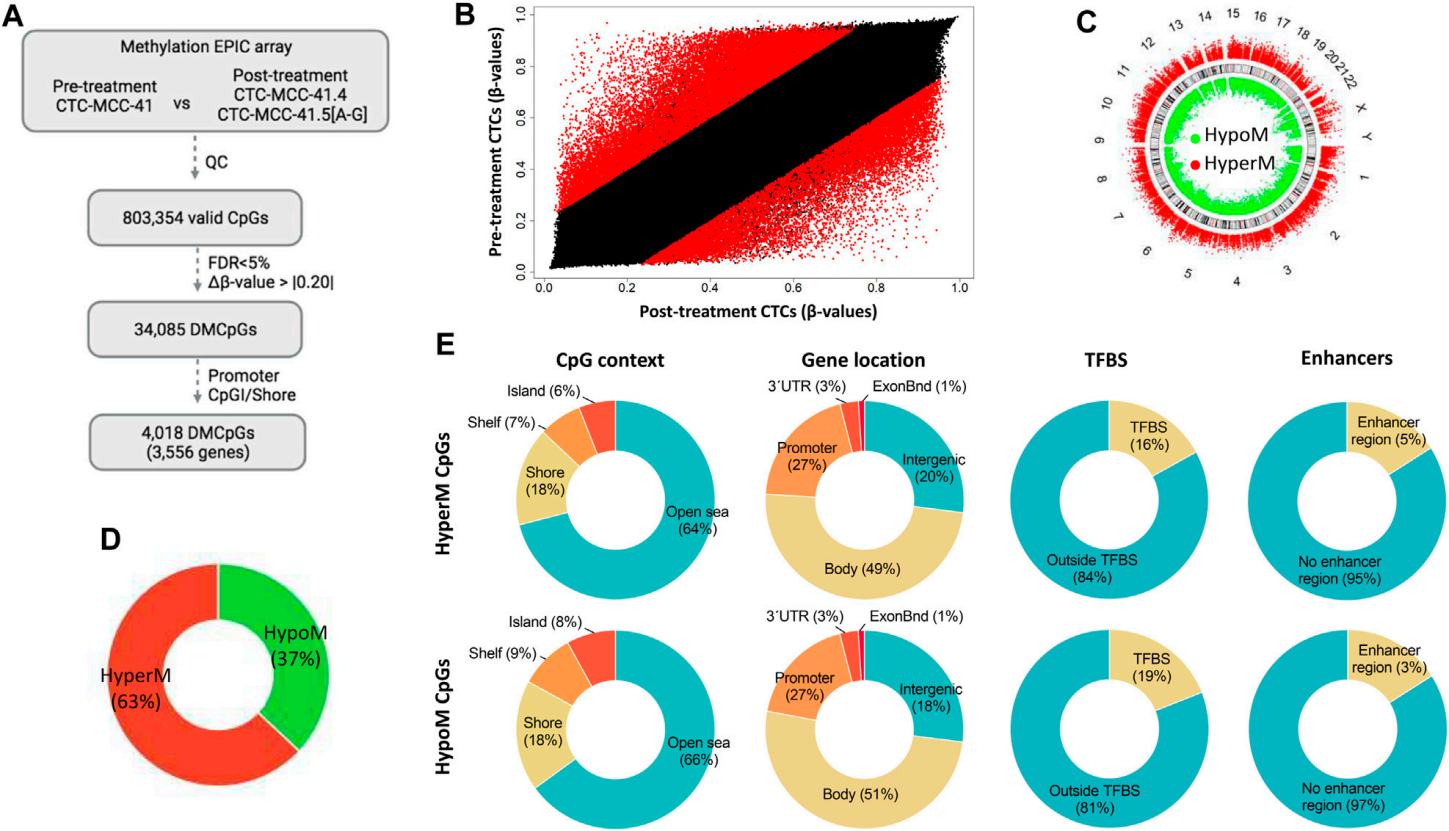
Genome-wide DNA methylation analysis of the pre-treatment CTC-MCC-41 line and the post-treatment CTC-MCC-41.4 and CTC-MCC-41.5 [A-G] lines. **(A)** Approach used to identify significant DMCpGs between the pre- and post-treatment CTC lines. **(B)** Scatter plot showing the mean normalized levels of DNA methylation (β-values) in the pre-treatment and post-treatment CTC lines. Red dots indicate DMCpGs. **(C–E)** Description of the 34,085 DMCpGs found in the pre-treatment CTC-MCC-41 line compared with the eight post-treatment CTC lines according to **(C)** their chromosome location, **(D)** global DNA methylation status, **(E)** CpG context (gene location, TFBS and enhancers). FDR, false discovery rate. CpGI, CpG island; ExonBnd, exon boundaries; HypoM, hypomethylated; HyperM, hypermethylated; QC, quality control; TFBS, transcription factor binding site.

The DNA methylation profiles of the pre-treatment CTC-MCC-41 line and of the eight post-treatment CTC lines (CTC-MCC-41.4 and CTC-MCC-41.5 [A-G]) were obtained using the EPIC array. After data normalization and quality control, 803,354 valid CpGs were selected for the analysis (Figure 1A). A scatter plot analysis revealed differences in the DNA methylation profiles of pre- and post-treatment CTC lines (Figure 1B). Specifically, this analysis identified 34,085 significantly differentially methylated CpGs (DMCpGs) (*p*-value <0.05; FDR adjusted *p*-value <0.05; Δβ-value >|0.20|) between CTC-MCC-41 and the eight post-treatment CTC lines, among which 4,018 DMCpGs were located in promoters and shore regions.

The 34,085 DMCpGs showed a wide distribution throughout the genome (Figure 1C). In pre-treatment CTC-MCC-41 cells, 63% of all DMCpGs (21,409 CpGs) were hypermethylated and 37% (12,676 CpGs) were hypomethylated compared with the post-treatment CTC lines (Figure 1D). Both hyper- and hypomethylated CpGs in CTC-MCC-41 cells showed a similar distribution (i.e., gene region location and CpG context) (Figure 1E). Most of these DMCpGs (64% of hyper- and 66% of hypo-methylated CpGs) were located in open sea regions, characterized by low CpG density. In gene regions, both hyper- and hypo-methylated CpGs were fairly homogeneously distributed throughout promoters, gene bodies, and intergenic regions. Moreover, DNA methylation differences were observed between CTC-MCC-41 and the post-treatment CTC lines in CpGs located at transcription factor binding sites (TFBS). Specifically, 2,369 CpGs were hypomethylated at TFBS (19% of all hypomethylated CpGs) and 3,591 CpGs were hypermethylated at TFBS (16% of all hypermethylated CpGs) in the CTC-MCC-41 line compared with the other CTC lines. Moreover, a small percentage of DMCpGs (5% of hyper- and 3% of hypo-methylated CpGs in CTC-MCC-41 line) was located at enhancer regions.

**FIGURE 2:**
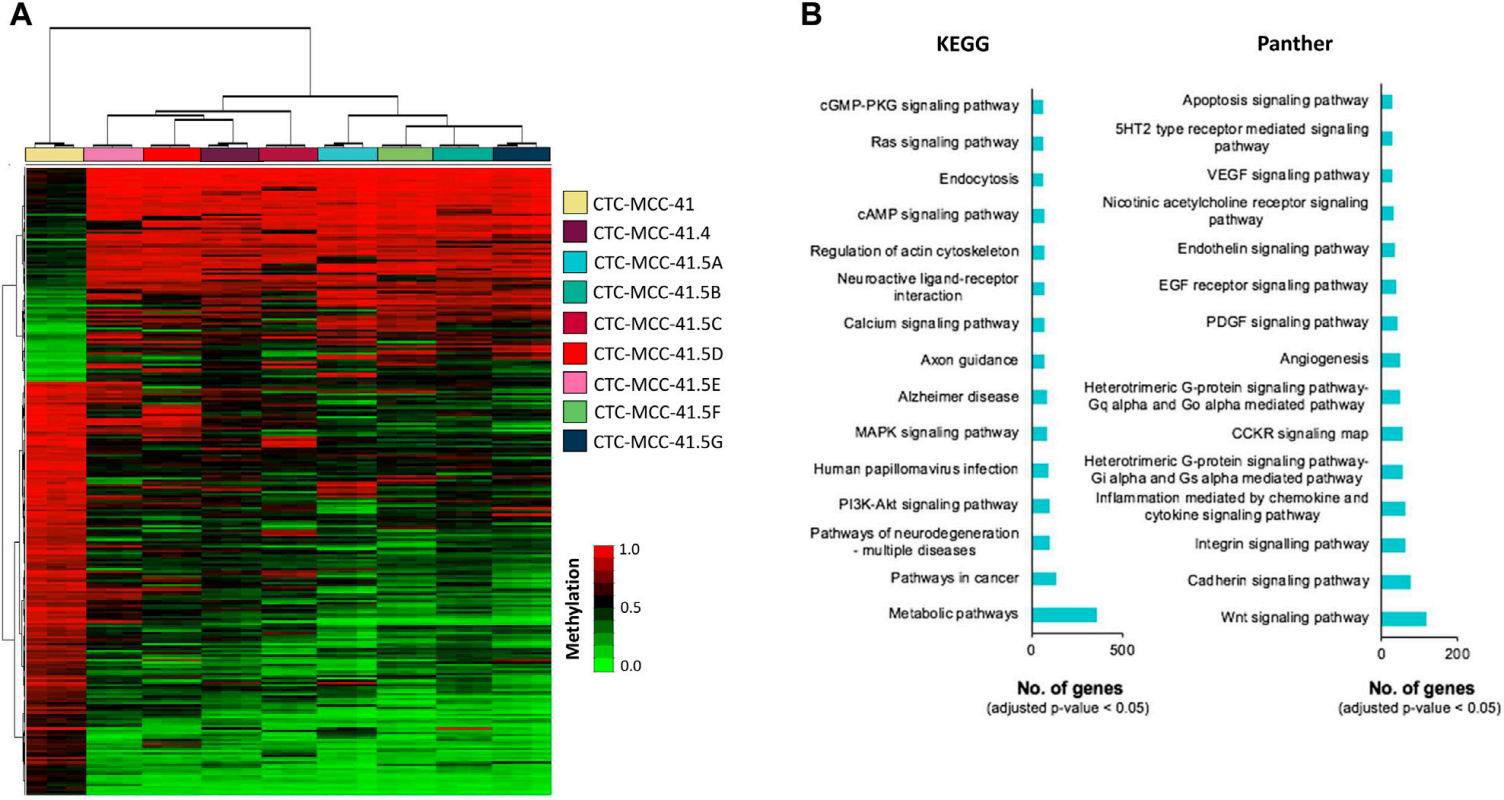
DNA methylation signature of the pre-treatment CTC-MCC-41 line compared with the post-treatment CTC-MCC-41.4 and CTC-MCC-41.5 [A-G] lines. **(A)** Hierarchical clustering heatmap of the 10,000 most DMCpGs (FDR adjusted *p*-value <0.05) in CTC-MCC-41 cells and all eight post-treatment cell lines. **(B)** GO analysis showing some of the most cancer-relevant KEGG and Panther pathways based on the 10,000 most DMCpGs in the pre-treatment CTC line compared with all post-treatment CTC lines.

A hierarchical clustering analysis using the 10,000 most differentially methylated CpGs showed that the DNA methylation profile could be used to clearly differentiate the pre- and post-treatment CTC lines (Figure 2A). The hierarchical clustering analysis also separated the post-treatment CTC lines in two branches: i) CTC-MCC-41.4 and CTC-MCC-41.5 [CDE] and ii) CTC-MCC-41.5 [ABFG]. Supplementary Table S3 lists the 20 top DMCpGs between pre- and post-treatment CTC lines. KEGG and Panther analyses revealed that the obtained methylation profile was enriched in pathways related to PI3K/AKT and MAPK signaling, metabolism, Wnt signaling, cadherin and integrin signaling, inflammation and apoptosis, among others (Figure 2B).

### 3.2 Comparison of the DNA methylation profiles of post-treatment CTC lines

**FIGURE 3:**
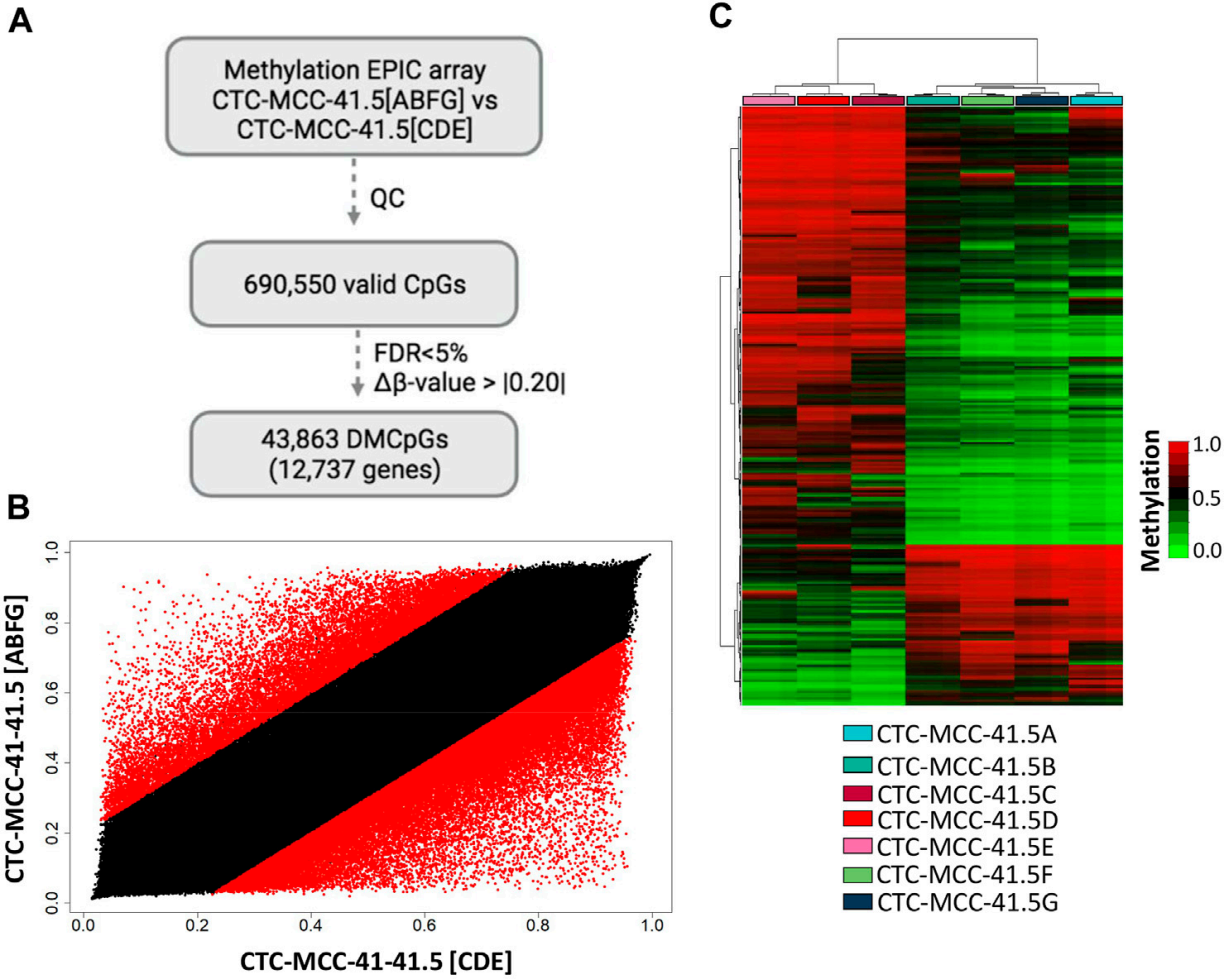
Genome-wide DNA methylation analysis of the post-treatment CTC-MCC-41.5 [ABFG] lines and CTC-MCC-41.5 [CDE] lines. **(A)** Approach used to identify significant DMCpGs in the CTC-MCC-41.5 [ABFG] lines compared with the CTC-MCC-41.5 [CDE] lines. **(B)** Scatter plot showing the mean normalized DNA methylation levels (β-values) in the CTC-MCC-41.5 [ABFG] and CTC-MCC-41.5 [CDE] lines. Red dots indicate significant DMCpGs. **(C)** Hierarchical clustering heatmap of the 10,000 most DMCpGs (FDR adjusted *p*-value <0.05) in the CTC-MCC-41 [ABFG] lines compared with the CTC-MCC-41 [CDE] lines. QC, quality control; FDR, false discovery rate.

First, the DNA methylation profiles of the CTC-MCC-41.4 (derived from a blood sample collected at the time of the second relapse at the end of second-line therapy) and the CTC-MCC-41.5 [A-G] (derived from a blood sample collected after the last cancer relapse, 1 week before the patient death) lines were compared (Soler et al., 2018). This comparison identified only 4,475 DMCpGs (*p*-value <0.05; FDR adjusted *p*-value <0.05; Δβ-value >|0.20|) (Supplementary Figure S1A). Similarly, the scatter plot analysis revealed rather similar methylation patterns between the CTC-MCC-41.4 and the CTC-MCC-41.5 [A-G] lines (Supplementary Figure S1B). As our previous analysis revealed different DNA methylation profiles between the CTC-MCC-41.5 [ABFG] lines and the CTC-MCC-41.5 [CDE] lines (Figure 2A), the methylome of these two CTC groups were compared. This led to the identification of 43,863 DMCpGs (*p*-value <0.05; FDR adjusted *p*-value <0.05; Δβ-value >|0.20|) between the CTC-MCC-41.5 [ABFG] and CTC-MCC-41.5 [CDE] groups (Figure 3A). The scatter plot analysis also revealed a different DNA methylation pattern between these CTC groups (Figure 3B). The hierarchical clustering analysis of the 10,000 most DMCpGs between these groups revealed DNA methylation profiles that clearly differentiated the CTC-MCC-41.5 [ABFG] from the CTC-MCC-41.5 [CDE] lines (Figure 3C).

### 3.3 Association of the DNA methylation and transcriptional profiles in the pre- and post-treatment CTC lines

**FIGURE 4:**
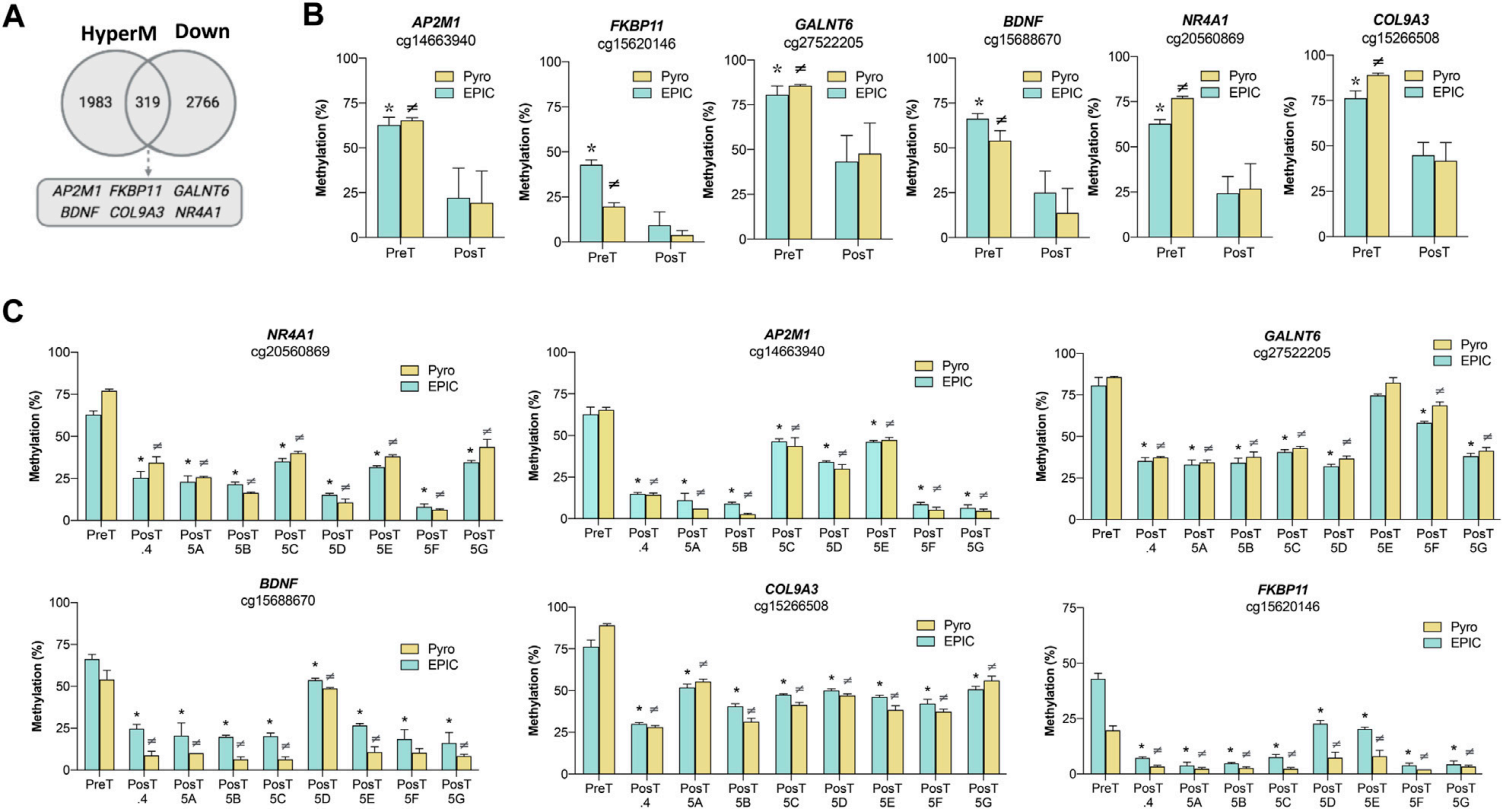
Effect of DNA methylation changes on the transcriptional profile of the pre-treatment CTC-MCC-41 line and the post-treatment CTC-MCC-41.4 and CTC-MCC-41.5 [A-G] lines. **(A)** Association between DNA hypermethylation and gene downregulation in the pre-treatment CTC-MCC-41 line compared with all eight post-treatment CTC lines (CTC-MCC-41.4 and CTC-MCC-41.5 [A-G]). **(B,C)** Validation of the DNA methylation status of representative promoter CpGs at selected genes in the pre-treatment CTC-MCC-41 line **(B)** compared with all eight post-treatment cell lines globally and **(C)** individually. EPIC array data are expressed as percentage (%) of DNA methylation. *, *p*-value <0.05 between the pre-treatment CTC-MCC-41 and the post-treatment CTC lines after EPIC array analysis; ≠, *p*-value <0.05 between the pre-treatment CTC-MCC-41 and post-treatment cell lines after pyrosequencing analysis. HyperM, hypermethylated; Down, downregulated. Pyro, pyrosequencing; Post.4, Post-treatment CTC-MCC-41.4 line; Post 5 [A-G], Post-treatment CTC-MCC-41.5 [A-G] lines.

The transcriptional profiles of the pre-treatment CTC-MCC-41 and the post-treatment CTCs (CTC-MCC-41.4 and CTC-MCC-41.5 [A-G]) lines were analyzed in a previous work (Cayrefourcq et al., 2021), revealing 6,327 differentially expressed transcripts between these CTC groups (1,857 up- and 4,470 downregulated in CTC-MCC-41 respect to the post-treatment CTCs). These expression data were used in this study to evaluate the association of gene expression with the DNA methylation changes observed in CpGIs and shore regions of promoters. Particularly, as the CTC-MCC-41 line was more hypermethylated than the post-treatment CTC lines, this analysis focused on associations between DNA hypermethylated and downregulated genes in CTC-MCC-41 cells (Figure 4A). The Venn diagram showed that in the pre-treatment CTC-MCC-41 line, 319 genes were hypermethylated and transcriptionally silenced. Six of these genes (*AP2M1*, *FKBP11*, *GALNT6*, *BDNF*, *COL9A3* and *NR4A1*) were selected for validation based on the following criteria: i) high DNA methylation (Δβ-value ≥0.30; Supplementary Table S4) and high gene expression difference (fold change ≥2.0) between the CTC-MCC-41 and the post-treatment CTC lines; and ii) implication in cancer-relevant pathways. For all the CpGs and genes analyzed, the pyrosequencing analysis showed higher DNA methylation levels in the pre-treatment CTC-MCC-41 line than in all post-treatment CTC lines (Figure 4B). Similar results were obtained when the DNA methylation of each post-treatment CTC line was analyzed individually (Figure 4C). The negative association between the methylation and expression of all the selected genes was validated by RT-qPCR analysis, by comparing the gene expression from three hypomethylated post-treatment CTC lines with the hypermethylated CTC-MCC-41 line. As expected, all the genes showed significantly higher expression in the hypomethylated post-treatment cell lines than in the hypermethylated pre-treatment cell line (Supplementary Figure S2). Altogether, these results confirm that the genes *AP2M1*, *FKBP11*, *GALNT6*, *BDNF*, *COL9A3*, and *NR4A*, are epigenetically regulated by methylation changes in the CTC lines analyzed.

### 3.4 Epigenetic regulation of *AP2M1* in the pre- and post-treatment CTC lines

**FIGURE 5:**
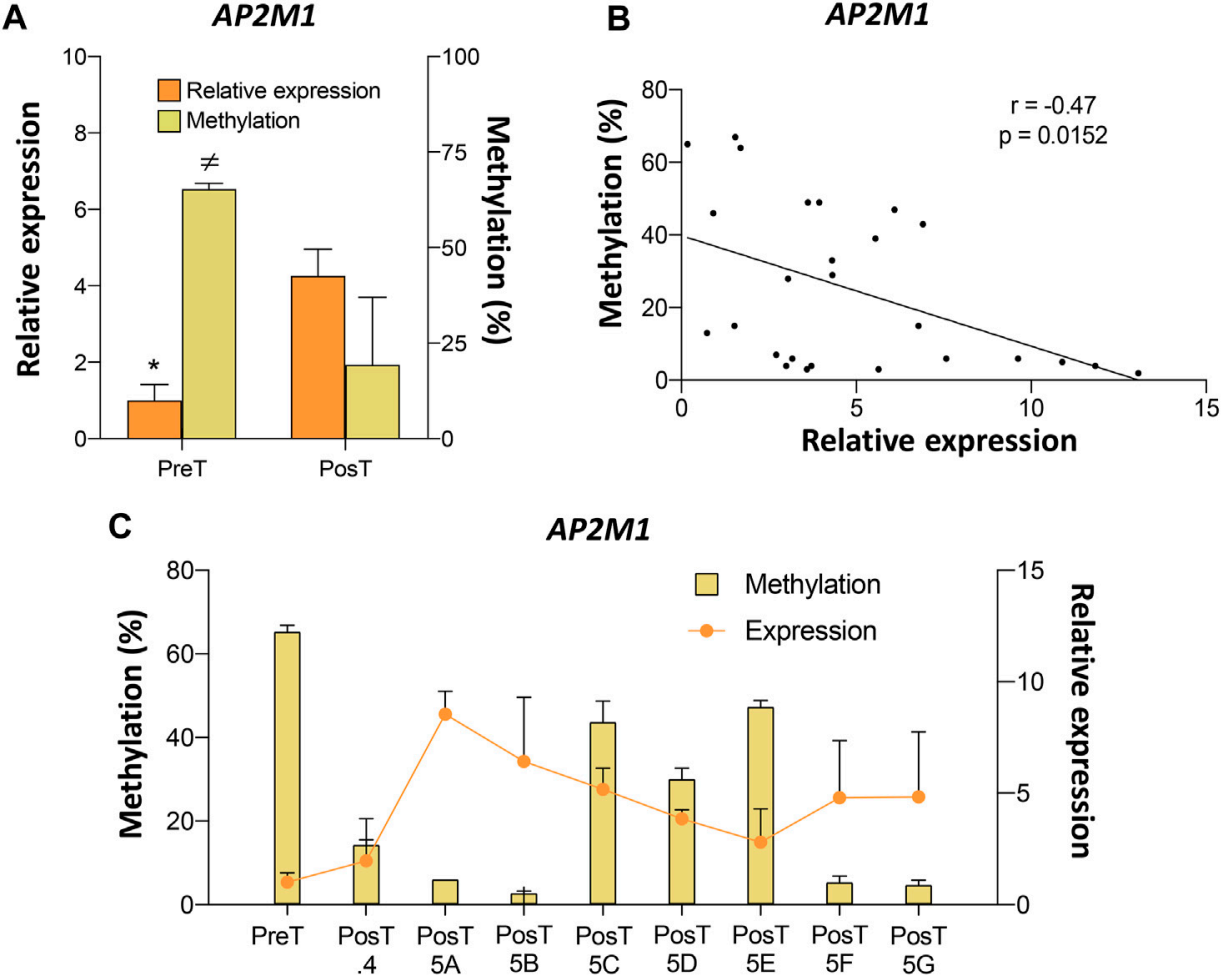
Epigenetic regulation of the *AP2M1* gene in the pre-treatment CTC-MCC-41 and post-treatment CTC-MCC-41.4 and CTC-MCC-41.5 [A-G] lines. **(A)** Levels of DNA methylation at the *AP2M1* promoter and *AP2M1* expression in the pre-treatment CTC-MCC-41 line compared with all eight post-treatment cell lines. *, *p*-value <0.05 for gene expression between the pre-treatment CTC-MCC-41 line and all eight post-treatment cell lines; ≠, *p*-value <0.05 for DNA methylation between the pre-treatment CTC-MCC-41 line and all eight post-treatment cell lines. **(B)** Correlation between DNA methylation and expression levels of *AP2M1* in the pre-treatment CTC-MCC-41 and all eight post-treatment CTC lines. **(C)** DNA methylation level at the promoter and *AP2M1* expression in the pre-treatment CTC-MCC-41 line and in each post-treatment CTC line. PreT, pre-treatment CTC line; PosT, all eight post-treatment CTC lines; PosT.4, post-treatment CTC-MCC-41.4 line; PosT5 [A-G], post-treatment CTC-MCC-41.5 [A-G] lines. Methylation (cg14663940) and expression values were determined in triplicates by pyrosequencing and RT-qPCR, respectively, and are expressed as mean ± SD.

Among the genes validated by pyrosequencing, *AP2M1* showed the highest DNA methylation differences between pre- and post-treatment CTC lines (Supplementary Table S4). Analysis of the pre- and post-treatment CTC lines revealed a negative association between the DNA methylation levels at the *AP2M1* promoter and its expression level (Figure 5A). Moreover, a negative correlation between DNA methylation and *AP2M1* expression was also observed when the paired values of the pre-treatment line and all post-treatment CTC lines were considered (Figures 5B, C). Among all post-treatment CTC lines, the CTC-MCC-41.5 [ABFG] lines were characterized by the lowest DNA methylation level at the *AP2M1* promoter.

## 4 Discussion

Epigenomic reprogramming is a hallmark of cancer that can induce broad epigenetic shifts in tumors (Hanahan, 2022) and promote the development of drug-resistance (Sawant et al., 2023). Understanding the underlying mechanisms of resistance and identifying new biomarkers are necessary steps to improve the outcome of patients with CRC (Luo et al., 2021). To this aim, the molecular characterization of CTC lines has proven to be a relevant approach (Cayrefourcq et al., 2021). Here, we compared the DNA methylome of the pre-treatment CTC line CTC-MCC-41 and of eight post-treatment CTC lines (CTC-MCC-41.4 and CTC-MCC-41.5 [A-G]) that were established from blood samples of a patient with mCRC during disease progression and development of resistance to 5-fluorouracile-based therapy. This comparison showed that the DNA methylation profile of metastasis-competent CTCs is reprogrammed during cancer progression and therapy resistance development. These epigenetic changes were able to modulate the transcription process, restoring the expression of genes with pro-tumoral functions through hypomethylation. These results suggest that the epigenomic reprogramming observed in resistant CTCs could be a mechanism to favor the pro-tumoral properties of these cells and escape the action of the treatment, which would have negative consequences for the outcome of the disease. The epigenetically deregulated genes identified could also have impact in the clinic as non-invasive biomarkers for therapy resistance and novel therapeutic targets for CRC patients.

In our study, the post-treatment CTC lines derived during therapy resistance development showed a different global DNA methylation profile compared with the pre-treatment CTC line. Specifically, we observed a generalized loss of DNA methylation in the post-treatment CTC lines, suggesting that the selective treatment pressure can alter the DNA methylome of CTCs, leading to a more hypomethylated profile. The DNA methylation differences were mainly observed at CpG-poor regions. This suggests that the epigenetic deregulation of these genomic regions may play an important role in the acquisition of therapy resistance. Typically, tumors exhibit hypomethylation of CpG-poor regions that normally comprise the majority of the methyl-cytosine content (Feinberg et al., 1988). This hypomethylation has been associated with the development of chromosomal instability (CIN), proto-oncogene expression, mismatch repair deficiency (MMRd) and the derepression of transposable elements, leading to malignant transformation of tumors (Esteller, 2008; Sheaffer et al., 2016; Besselink et al., 2023). Among these alterations, CIN has been recently proposed as one of the most relevant consequences of the generalized DNA methylation loss (Besselink et al., 2023). Hypomethylation induced CIN promotes tumor heterogeneity and cancer evolution (Besselink et al., 2023), as well as accelerates the acquisition of drug resistance through the gain of aneuploidies (Lukow et al., 2021), indicating that the DNA methylation loss observed in CTCs under selective treatment pressure could be a mechanism of therapy resistance. From a clinical point of view, analyses of hypomethylated regions in tumors of colon cancer patients have proven to be useful as biomarkers in different contexts (Tang et al., 2015), indicating that the discovery of hypomethylated regions in resistant CTCs could be useful as potential biomarkers of therapy resistance. In addition to CpG-poor regions, some of the DNA methylation changes that we observed in resistant CTCs were located in CpGIs, shores and promoters. In line with this, previous studies also reported DNA methylation changes at gene promoters in CRC cells associated with the development of chemotherapy resistance (Zhao et al., 2015; Wen et al., 2018).

We also identified a distinctive DNA methylation signature that clearly differentiated the pre-treatment CTC line from the post-treatment CTC lines. The genes in this signature were associated with relevant cancer pathways, including PI3K/AKT, MAPK, Wnt signaling and metabolism. The activation of PI3K/AKT and MAPK pathways are critical to induce anti-apoptosis activity and increase the aggressiveness of colon cancer cells resistant to 5-fluorouracile-based therapy (Huang et al., 2016). The Wnt signaling is one of the main pathways regulating stemness in cancer cells (Vermeulen et al., 2010) and control cell proliferation, survival, migration, and invasiveness. Alterations of this pathway are able to confer chemoresistance by maintaining the cancer stem cell population, favoring transcriptional plasticity, improving DNA damage repair, or inducing immune evasion (Moreno-Londono et al., 2023). The deregulation of metabolic pathways in cancer cells is also often involved in the development of drug resistance. For example, uncontrolled glycolysis or abnormally activated glycolysis (e.g., Warburg effect) is usually linked to oncogenesis and chemoresistance (Liu et al., 2021). Therefore, the epigenetic deregulation of these pro-tumoral pathways in CTCs during treatment pressure could provide the CTCs with new properties to induce resistance, proliferate and escape the action of therapy. In line with our results, a recent work described the transcriptional deregulation of these pathways in the pre-treatment CTC-MCC-41 line (Smit et al., 2020) and another study identified Wnt/beta-catenin signaling as the final step of transcriptional activation in these CTCs (Zhou et al., 2023). Moreover, other authors have described the molecular deregulation of these pathways in association with proliferation and drug resistance in CRC (Stefani et al., 2021; Lin et al., 2022).

Conversely, we did not find many DNA methylation differences between the post-treatment CTC-MCC-41.4 (obtained after the last treatment) and CTC-MCC-41.5 [A-G] (obtained before the patient´s death) lines. Similarly, a previous work found very few differences in gene expression among these cell lines, indicating that, in the absence of treatment pressure, the worsening of cancer is no longer linked to clonal evolution but rather to the natural progression of the disease through the replication of the CTC clones already selected under treatment (Cayrefourcq et al., 2021). However, DNA methylation presented many differences between the CTC-MCC-41.5 [ABFG] and CTC-MCC-41.5 [CDE] lines, and a DNA methylation signature could clearly differentiate these two groups. This result agrees with previous gene expression data on these CTC lines that could be used to differentiate these two groups. In particular, in this previous study, the comparison of the gene expression signatures of the two CTC-MCC-41.5 sub-groups ([ABFG] and [CDE]) revealed that *ALDOB* was upregulated in the [ABFG] group, which is mainly expressed in the liver, suggesting that the subgroup [ABFG] derives from CTCs released by liver metastases (Cayrefourcq et al., 2021). According to this, the different origin of the release of CTCs could contribute to explain the molecular differences observed between the subgroups [ABFG] and [CDE].

In this work, we also observed that DNA methylation changes in post-treatment CTC lines were associated with changes in gene expression. For example, *AP2M1*, *FKBP11*, *GALNT6*, *BDNF*, *COL9A3* and *NR4A1* showed a negative association between promoter DNA methylation and gene expression. These genes were DNA hypomethylated and upregulated in the resistant clones (CTC-MCC-41.4 and CTC-MCC-41 [A-G]), suggesting that the selective treatment pressure can restore their expression by modulating their epigenetic program. In line with this, the increased expression of these pro-tumoral genes was previously associated with the development of therapy resistance. *BDNF* upregulation in CRC has been linked to enhanced dissemination, anoikis resistance, and chemoresistance (Ha et al., 2021). In neuroblastoma, *BDNF* expression protects cells from chemotherapy-induced apoptosis via PI3K and also increases metastasis formation via the PI3K and MAPK pathways (Jaboin et al., 2002; Hua et al., 2016). *FKBP11* expression is strongly correlated with advanced disease and poor prognosis in patients with renal cancer (Li et al., 2022), and can promote cell proliferation and tumorigenesis (Qiu et al., 2021). Moreover, *COL9A3* promotes epithelial-to-mesenchymal transition, invasion, and migration in gastric cancer (Wu et al., 2021). *GALNT6* overexpression has been associated in CRC with resistance to fluoropyrimidines and CRC cell proliferation and migration (Peng et al., 2021). This gene is also involved in the activation of the PI3K/AKT signaling pathway and in breast cancer cell migration and invasion (Liu et al., 2020). *NR41A* is implicated in cancer progression by promoting metastasis, inflammation, proliferation, stemness and epithelial-to-mesenchymal transition and by inhibiting apoptosis (Shi et al., 2021). *AP2M1* showed an inverse correlation between DNA methylation and expression in the pre- and post-treatment CTC lines. This gene encodes a subunit of the hetero-tetrameric coat assembly protein complex 2 (AP2) that is required for vacuolar ATPase activity and is involved in regulating protein intracellular trafficking (Liu et al., 2023). Increased *AP2M1* activity has been associated with chemotherapy resistance in several tumor types, including lymphoma (Liu et al., 2023), breast cancer and CRC (Le Duff et al., 2018). Chemotherapy acts also by inducing cancer cell senescence and growth arrest and by inhibiting proliferation. However, cancer cells may acquire new properties, escape senescence, and start to proliferate again (Le Duff et al., 2018; Guillon et al., 2019). It has been recently shown that *AP2M1* participates in the transmission of soluble signals from senescent CRC cells to promote resistance to chemotherapy (Le Duff et al., 2018), suggesting that *AP2M1* upregulation by DNA hypomethylation could be a novel mechanism by which CTCs escape from chemotherapy. In addition, the lowest DNA methylation level at the *AP2M1* promoter was observed in the resistant CTC-MCC-41.5 [ABFG] clones. Recently, it has been proposed that the CTC-MCC-41.5 [ABFG] clones are released by liver metastases (Cayrefourcq et al., 2021), suggesting that the DNA hypomethylation of the *AP2M1* promoter could be a relevant mechanism of resistance in CTCs originating from liver metastases. All these genes (*AP2M1*, *FKBP11*, *GALNT6*, *BDNF*, *COL9A3* and *NR4A1*) favor relevant features of cancer progression, indicating that their epigenetic activation in CTCs of CRC patients under treatment could be a key event for the development of therapy resistance and cancer progression. Among the clinical applications of DNA methylation in cancer, the evaluation of this epigenetic mark in CTCs has been recently suggested as a non-invasive biomarker for monitoring the evolution of the disease during treatment and detecting resistance in lung cancer (Ntzifa et al., 2021). In line with this, analysis of CTCs to monitor the loss of methylation in the genes identified in our work could be useful in the clinic to detect the development of therapy resistance and the progression of the disease in CRC patients. This potential clinical application should be confirmed in future clinical studies.

Loss of methylation in tumors is able to restore the expression of oncogenes that had been epigenetically silenced (Lu et al., 2020). The action of drugs directed to oncogenic proteins represent a key strategy to fight against cancer. For example, the use of therapeutic targets against EGFR (cetuximab) and VEGF (bevacizumab) has shown efficacy to treat mCRC patients (Culy, 2005; Tabernero et al., 2007). Importantly, targeting oncogenes in CTCs is able to reduce the tumoral properties of these cells. A recent *in vitro* study demonstrated that AKT and mTOR inhibitors are able to reduce the growth of CTCs (Smit et al., 2020). In our study, loss of methylation in resistant CTCs restored the expression of several genes with pro-tumoral properties, suggesting that targeting these genes in CTCs could be a new approach to combat the acquisition of therapy resistance and progression in colon cancer.

In summary, this study shows that therapy pressure can induce a global reprogramming in the DNA methylation profile of CTCs, leading to a more hypomethylated profile. Loss of methylation in CTCs during selective treatment pressure could represent a novel mechanism for inducing treatment resistance. Therapy-resistant CTCs also showed a distinctive DNA methylation signature enriched in relevant cancer pathways related to the acquisition of therapy resistance and tumor progression. Several epigenetically deregulated genes associated with therapy resistance in CTCs were identified, particularly *AP2M1*. Loss of methylation in CTCs could be a potential non-invasive biomarker in CRC patients for monitoring the effectiveness of treatment and the development of resistance, which should be evaluated and confirmed in future clinical studies. Thus, the genes identified in this study represent new potential biomarkers of resistance and therapeutic targets that could contribute to the development of new clinical tools and therapies for CRC patients. The findings of this work open new avenues for future studies that evaluate the reprogramming of CTCs as a mechanism of therapy resistance in cancer, with potential biological and clinical implications for the progression of the disease.

## Data Availability

The data presented in the study are deposited in the Gene Expression Omnibus (GEO) repository, accession number GSE239549.
