# Supplementary material for: Epigenomic reprogramming of therapy-resistant circulating tumor cells in colon cancer
Source: Front Cell Dev Biol. 2023 Dec 21;11:1291179. doi: 10.3389/fcell.2023.1291179 (PMC10771310; doi:10.3389/fcell.2023.1291179)
Supplement: Supplementary file 1 [file DataSheet1.PDF]

Supplementary Material

Epigenomic reprogramming of therapy-resistant circulating tumor cells in colon cancer

Aida Bao-Caamano, Nicolás Costa-Fraga, Laure Cayrefourcq, Aitor Rodriguez-Casanova, Laura Muinelo-Romay, Rafael López-López*, Catherine Alix-Panabières*, Angel Díaz-Lagares*

*** Correspondence:** Angel Díaz-Lagares: angel.diaz.lagares@sergas.es; Catherine Alix-Panabières: c-panabieres@chu-montpellier.fr; Rafael López-López: rafael.lopez.lopez@sergas.es

1 Supplementary Figures and Tables

1.1 Supplementary Figures

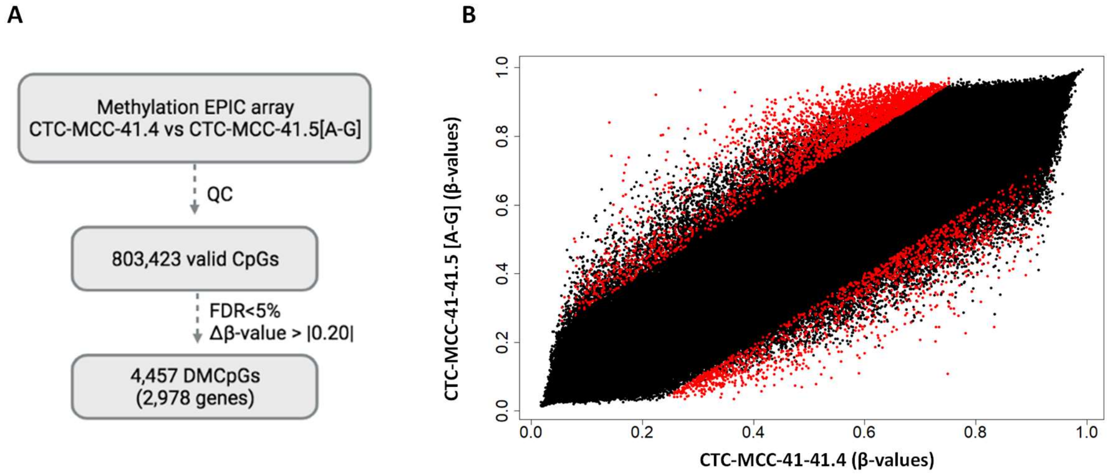

Supplementary Figure 1. Genome-wide DNA methylation analysis of the post-treatment cell lines CTC-MCC-41.4 respect to CTC-MCC-41.5 [A-G]. (**A**) Schematic flowchart used to identify significant DMCpGs in CTC-MCC-41.4 compared to CTC-MCC-41.5 [A-G]. (**B**) Scatter plot representing mean normalized levels of DNA methylation (β-values) in CTC-MCC-41.4 and CTC-MCC-41.5 [A-G]. Dots in red show significant DMCpGs. QC, quality control; FDR, false discovery rate.

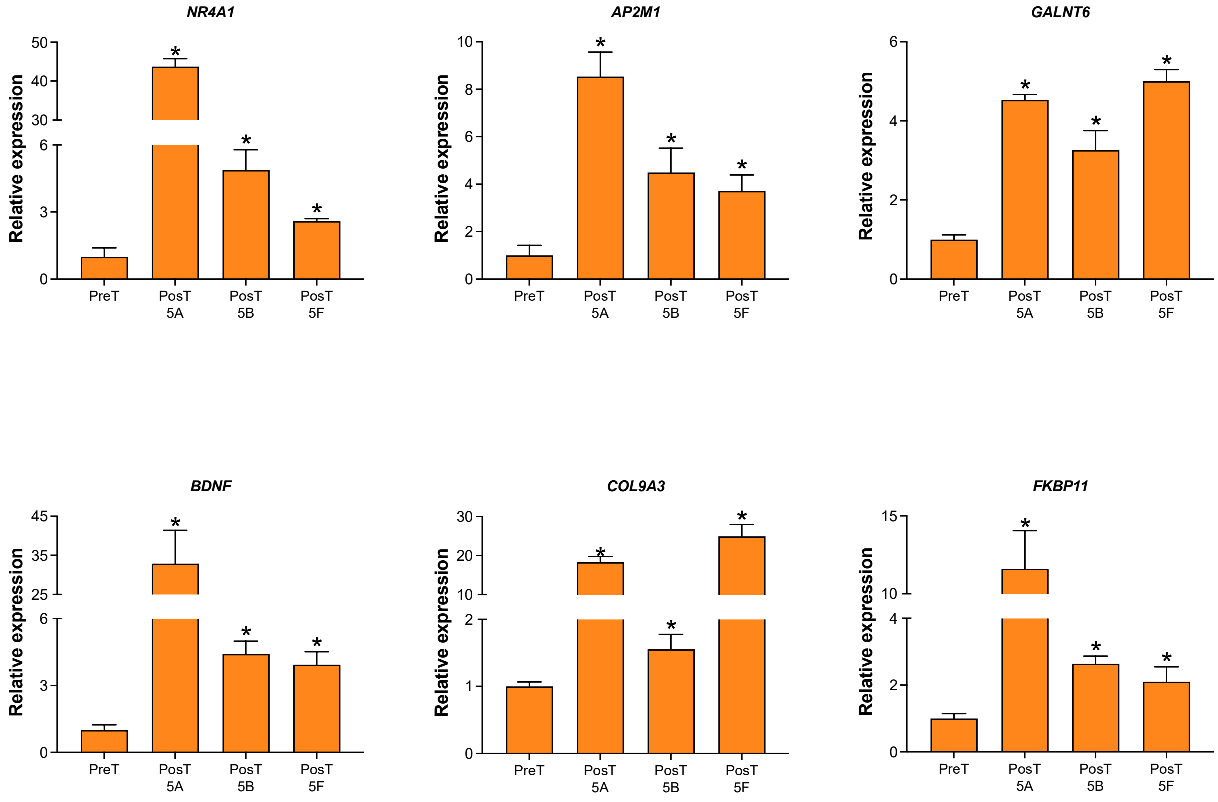

Supplementary Figure 2. Gene expression levels of selected genes in hypomethylated post-treatment cell lines respect to the hypermethylated pre-treatment CTC-MCC-41. PreT, pre-treatment CTC-MCC-41; PosT5A, post-treatment CTC-MCC-5A; PosT5B, post-treatment CTC-MCC-5B; PosT5F, post-treatment CTC-MCC-5F. Expression values were determined in triplicates by RT-qPCR and are expressed as mean ± SEM. *, p-value <0.05 between the pre-treatment CTC-MCC-41 and each post-treatment CTC line.

1.2 Supplementary Tables

Supplementary Table 1. Primer sequences for DNA methylation analysis by pyrosequencing.

Gene	Primer	Sequence (5´- 3´)
AP2M1	Forward	TGTTTGGGGGTTTTAGGTTTGAATTA
	Reverse	[Btn] CTCAACCAAACTCCAATTCCTATTTATTC
	Sequencing	AGGTAAGTTTTAGTGAGTTAT
BDNF	Forward	GGGGTTGGAAGTGAAAATATTTGTAAAAG
	Reverse	[Btn] ACCCCATCAACRAAAAACTCCATT
	Sequencing	GGTAGYGGAGGTATTATATGATAG
COL9A3	Forward	TTTTTGGGAAATGGATAT
	Reverse	[Btn] TCAAACCACRTCAATTACTA
	Sequencing	ATTTYGGGGAGGGGT
FKBP11	Forward	GGAGGAAGGGTTGTAGGA
	Reverse	[Btn] TCACCAAAATCTCCACTTAA
	Sequencing	GTTTAGTTTGGTTTGGG
GALNT6	Forward	AGGTATTGGGTGAGGTTT
	Reverse	[Btn] TTCCCTATCTCATCCTTCTT
	Sequencing	ATAGTTTTGGATATATTAAAGAGT
NR4A1	Forward	TAGGGAAGGTTTTAGGTG
	Reverse	[Btn] ATACCCCTCCCCCTAT
	Sequencing	GTTTGTGTTTAGTAGGGT

Btn, biotin.

Supplementary Table 2. Primer sequences for the RT-qPCR analysis.

Gene	Primer	Sequence (5´- 3´)
AP2M1	Forward	AGATGACATCGGGAGGAAC
	Reverse	CCATCACGTCACACATCTTA
BDNF	Forward	AGAAAAAAACACACACACACAC
	Reverse	AGCCAGTAAAGCAATGACAA
COL9A3	Forward	GGCATTGACGGAGAAGCTG
	Reverse	GTCCAGTCAGACCATCCAC
FKBP11	Forward	ATACACTACACGGGAAGCTTGGTA
	Reverse	GAAGACTCTGCTCCAGACCT
GALNT6	Forward	TGGCCCAAGAGAAATCCT
	Reverse	TCAGGTCAGGAACAAACATC
NR4A1	Forward	AAGGAAGTTGTCCGAACAGA
	Reverse	AGCTCCTGGAACTTGGAGTA
B2M	Forward	GTCTTTCAGCAAGGACTGGTCT
	Reverse	TTACATGTCTCGATCCCACTTAAC

Supplementary Table 3. Top 20 differentially methylated CpGs identified in CTC-MCC-41 in comparison to all post-treatment CTC lines.

TargetID[1]	Chr[2]	Position	Gene name	Gene region	CpG context	Δβ[3]
cg01448115	12	130422172			Open Sea	0.826
cg17196033	19	35168591	*ZNF302;ZNF302*	1stExon;5'UTR;Body	Island	0.817
cg23352597	X	46435742	*CHST7*	3'UTR	Shore	0.812
cg20506380	1	158612442	*SPTA1*	Body	Open Sea	-0.801
cg15356425	11	128822408			Open Sea	0.799
cg05147578	22	18559240	*PEX26*	TSS1500	Shore	0.796
cg14753356	6	30720108			Open Sea	0.795
cg01201472	12	12764255	*CREBL2*	TSS1500	Shore	0.789
cg22032510	X	49649233			Open Sea	-0.788
cg15979885	13	113244675			Shelf	0.787
cg23366050	18	159793	*USP14*	Body	Shore	0.786
cg06384491	X	57163888	*SPIN2A;SPIN2A*	5'UTR;1stExon	Shore	-0.780
cg10675561	1	120052814	*HSD3B1*	Body	Open Sea	0.779
cg05158109	X	40592695	*MED14*	Body	Shore	0.777
cg11646050	X	16736350	*SYAP1*	TSS1500	Shore	-0.774
cg18241219	X	16463998			Open Sea	-0.774
cg17057190	19	2747608			Open Sea	0.770
cg05056349	2	207629824	*FASTKD2;MDH1*	TSS1500;Body	Shore	-0.765
cg02298765	19	11450741	*RAB3D*	TSS1500	Shore	0.763
cg04517512	1	218651707	*MIR548F3*	Body	Open Sea	0.763

[1]Identification of the CpG according to EPIC array; [2]Chromosome; [3]Δβ-values (β-value pre-treatment cell line CTC-MCC-41 - β-value all post-treatment cell lines). CpGs in the table are arranged according to their absolute Δβ-value.

Supplementary Table 4. DNA methylation levels of the significantly DMCpGs of the six selected genes hypermethylated and downregulated in CTC-MCC-41 in comparison to all post-treatment CTC lines analyzed by EPIC array.

TargetID[1]	Chr[2]	Position	Gene name	Gene region	CpG contex	Δβ[3]
**cg14663940**	**3**	**183894397**	***AP2M1***	**5'UTR**	**Shore**	**0.406**
cg00260810	3	183894469	*AP2M1*	5'UTR	Shore	0.537
cg15914769	11	27722774	*BDNF;BDNF;BDNF;BDNF*	TSS200;TSS1500;Body;5'UTR	Shore	0.341
**cg15688670**	**11**	**27723190**	***BDNF;BDNF;BDNF;BDNF***	**TSS1500;Body;5'UTR;TSS200**	**Shore**	**0.413**
cg23497217	11	27723214	*BDNF;BDNF;BDNF;BDNF*	TSS1500;Body;5'UTR;TSS200	Shore	0.201
cg06816235	11	27742219	*BDNF;BDNF;BDNF;BDNF*	Body;5'UTR;TSS1500;1stExon	Island	0.368
cg16709385	20	61447581	*COL9A3*	TSS1500	Shore	-0.201
cg16461246	20	61447614	*COL9A3*	TSS1500	Shore	-0.228
cg12084392	20	61447623	*COL9A3*	TSS1500	Shore	-0.201
**cg15266508**	**20**	**61447742**	***COL9A3***	**TSS1500**	**Island**	**0.314**
cg16653692	20	61447749	*COL9A3*	TSS1500	Island	0.282
cg00289503	20	61470028	*COL9A3*	Body	Shore	-0.256
cg05883907	12	49318487	*FKBP11*	Body;Body;5'UTR	Island	0.430
cg23692087	12	49318557	*FKBP11*	Body;Body;5'UTR;1stExon	Island	0.224
cg22021756	12	49318740	*FKBP11*	Body;Body;TSS200	Island	0.210
cg19190217	12	49318749	*FKBP11*	Body;Body;TSS200	Island	0.292
**cg15620146**	**12**	**49318784**	***FKBP11***	**Body;Body;TSS200**	**Island**	**0.336**
cg01612057	12	49318847	*FKBP11*	Body;Body;TSS200	Island	0.335
cg23699916	12	49319263	*FKBP11*	1stExon;TSS1500;5'UTR;1stExon;5'UTR	Island	0.357

cg05999245	12	49319428	*FKBP11*	TSS200;TSS1500;TSS200	Island	0.241
**cg27522205**	**12**	**51784426**	***GALNT6***	**5'UTR**	**Shore**	**0.374**
cg25360385	12	51786547	*GALNT6*	TSS1500	Shore	0.233
cg12541478	12	52444020	*NR4A1*	TSS1500	Shore	0.373
cg17242930	12	52434102	*NR4A1;NR4A1*	Body;5'UTR	Shelf	0.508
**cg20560869**	**12**	**52447054**	***NR4A1;NR4A1***	**5'UTR;Body**	**Shore**	**0.386**

[1]Identification of the CpG according to EPIC array; [2]Chromosome; [3]Δβ-values (β-value pre-treatment cell line CTC-MCC-41 - β-value all post-treatment cell lines). All CpGs in the table are significant (FDR adjusted p-value <0.05) and are arranged according to the alphabetical order of the genes and the genomic position. CpGs selected for validation are indicated in bold.
